# Succinamide Derivatives Ameliorate Neuroinflammation and Oxidative Stress in Scopolamine-Induced Neurodegeneration

**DOI:** 10.3390/biom10030443

**Published:** 2020-03-13

**Authors:** Sumbal Iqbal, Fawad Ali Shah, Komal Naeem, Humaira Nadeem, Sadia Sarwar, Zaman Ashraf, Muhammad Imran, Tariq Khan, Tayyaba Anwar, Shupeng Li

**Affiliations:** 1 Department of Pharmaceutical Chemistry, Faculty of Pharmaceutical Sciences, Riphah International University, Islamabad 747424, Pakistan; sumbalqas@hotmail.com (S.I.); m.imran.khanzada@gmail.com (M.I.); tayyaba.anwar@riphah.edu.pk (T.A.); 2 State Key Laboratory of Oncogenomics, School of Chemical Biology and Biotechnology, Shenzhen Graduate School, Peking University, Shenzhen 518000, China; 3 Department of Pharmacology, Faculty of Pharmaceutical Sciences, Riphah International University, Islamabad 747424, Pakistan; fawad.shah@riphah.edu.pk (F.A.S.); komal.naeem@riphah.edu.pk (K.N.); 4 Department of Pharmacognosy, Faculty of Pharmaceutical Sciences, Riphah International University, Islamabad 747424, Pakistan; sadia.sarwar@riphah.edu.pk; 5 Department of Chemistry, Allama Iqbal Open University, Islamabad 747424, Pakistan; mzchem78@gmail.com; 6 Department of Pharmacy, Capital University of Science and Technology, Islamabad 747424, Pakistan; tariq.khan@cust.edu.pk

**Keywords:** neurodegeneration, succinamide derivatives, neuroprotective, scopolamine, cognitive impairment, molecular docking

## Abstract

Oxidative stress-mediated neuroinflammatory events are the hallmark of neurodegenerative diseases. The current study aimed to synthesize a series of novel succinamide derivatives and to further investigate the neuroprotective potential of these compounds against scopolamine-induced neuronal injury by in silico, morphological, and biochemical approaches. The characterization of all the succinamide derivatives was carried out spectroscopically via proton NMR (^1^H-NMR), FTIR and elemental analysis. Further in vivo experiments showed that scopolamine induced neuronal injury, characterized by downregulated glutathione (GSH), glutathione S-transferase (GST), catalase, and upregulated lipid peroxidation (LPO). Moreover, scopolamine increased the expression of inflammatory mediators such as cyclooxygenase2 (COX2), nuclear factor kappa B (NF-kB), tumor necrosis factor (TNF-α), further associated with cognitive impairment. On the other hand, treatment with succinamide derivatives ameliorated the biochemical and immunohistochemical alterations induced by scopolamine, further supported by the results obtained from molecular docking and binding affinities.

## 1. Introduction

Consistent reports have reiterated the involvement of inflammatory cascade and oxidative stress in the pathophysiology of neurodegenerative disorders including Alzheimer’s disease (AD), ischemic stroke, and Parkinson’s disease [1]. Neurodegenerative disorders contribute significantly to the global health burden and a substantial increase is estimated by 2050 [2]. The inflammatory mediators have a significant role in the progression of neuronal pathologies [3,4,5]. Furthermore, astrocytes and microglial cells are rapidly activated during neuronal insult, followed by brain translocation of granulocytes and leukocytes from circulating blood [6,7]. Moreover, mitochondrial dysfunction and oxidative phosphorylation decoupling generate numerous reactive oxygen species (ROS), leading to widespread oxidative stress [8]. ROS damage lipids, proteins and DNA, and cause intracellular organelle destruction via plasma and organelle membrane peroxidation [9]. These could further stimulate the release of biologically active free fatty acids, such as arachidonic acid and DNA fragmentation. Along with glutamate-induced excitotoxicity and inflammatory factors, the vicious cycles induced by ROS eventually activate the injury pathways and lead to cell necrosis and apoptosis [10].

Scopolamine is a non-selective muscarinic receptor antagonist, commonly employed to induce cognitive and memory impairment in experimental models of neurodegenerative disorders. Scopolamine is associated with elevated acetylcholinesterase activity, altered brain ROS levels, and increased expression of inflammatory mediators such as TNF-α and COX2, along with the attenuated level of an antioxidant such as GSH (glutathione) [11,12,13,14,15]. Previous studies demonstrated the hyperactivity of glial cells coupled to neuroinflammatory cascade in the scopolamine-induced memory impairment model [16].

Succinamide derivatives are a pharmacologically active class of nitrogen-containing heterocyclic compounds. Among them, ethosuximide, methsuximide, and phensuximide have shown anticonvulsant, neuroprotective, and antinociceptive properties and are useful for the treatment of various neuropsychiatric disorders. The convenient addition of a new functional group into the succinamide ring, which further modifies the spectroscopic and pharmacological properties of resultant derivatives, is a characteristic feature of succinamide [17]. Several strategies can be adopted to synthesize succinamide including multiple coupling agents. A remarkable milestone in this regard is to employ succinic anhydride, an amide bond precursor, for succinamide synthesis. Succinamide provided a marked improvement to both pharmacokinetics and pharmacodynamics as demonstrated previously [18]. A number of succinamide containing agents were reported for anti-inflammatory and anti-oxidant effects [19,20].

The current study was designed to synthesize a series of novel succinamide derivatives and to investigate their neuroprotective action in the scopolamine-induced neurodegenerative model. The results obtained will not only help us to understand the cascading mechanisms leading to cell death but will also provide a clue for succinamide derivatives to be a potential therapeutic in the future.

## 2. Materials and Methods

### 2.1. Drugs and Chemicals

Materials required for initiation were obtained from Sigma Aldrich (St. Louis, MO, USA). Melting points of all newly synthesized succinamide derivatives were recorded via the Digital Gallen Kamp apparatus (Sanyo, Osaka, Japan). A Bruker AM-300 (Billerica, Massachusetts, UK) in DMSO-*d*_6_ was employed to determine the proton NMR (^1^H-NMR) spectra at 300 MHz and TMS was used as an internal standard. FTIR spectra were recorded by using the FTIR spectrophotometer (ATR eco ZnSe, Vmax in cm^−1^). Elemental analysis was performed via LECO 183 CHNS analyzer (Changsha, Hunan, China). The monitoring of all chemical reactions was carried out by thin-layer chromatography (TLC). Antibodies (mouse anti-TNF-α, mouse anti-COX2), Avidin-Biotin Complex (ABC) Elite kit, 3,3-diaminobenzidine peroxidase were purchased from Santa Cruz Biotechnology (Dallas, TX, USA). Hydrogen peroxide, formalin, 1-chloro-2,4-dinitrobenzene, trichloroacetic acid, glutathione (GSH), thiobarbituric acid, and N-(1-naphthyl) ethylenediamine dihydrochloride were purchased from Sigma Aldrich (St. Louis, MO, USA). Secondary antibody goat anti-mouse was procured from Abcam (Cambridge, UK). All solvents, chemicals and reagents used were of 99% HPLC grade.

### 2.2. General Procedure for One-Pot Synthesis of Succinamide Derivatives(2a-2i)

**Figure 1:**
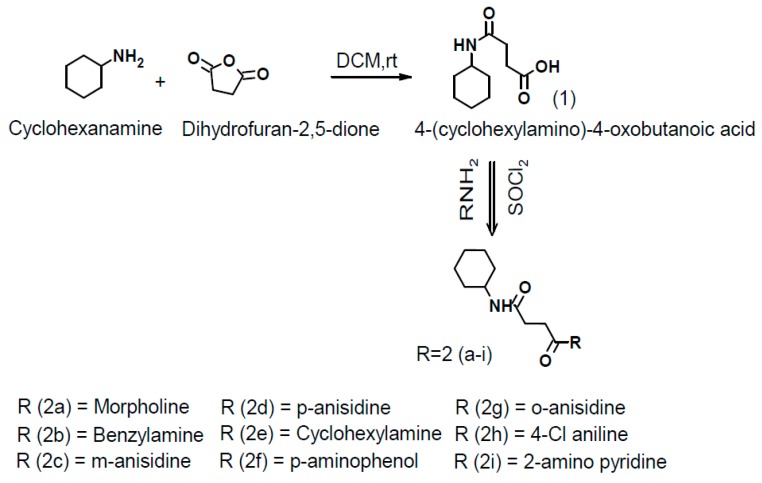
Scheme for the synthesis of new succinamide derivatives. DCM, dichloromethane; rt, room temperature; SOCl_2_, thionyl chloride.

**Figure 2:**
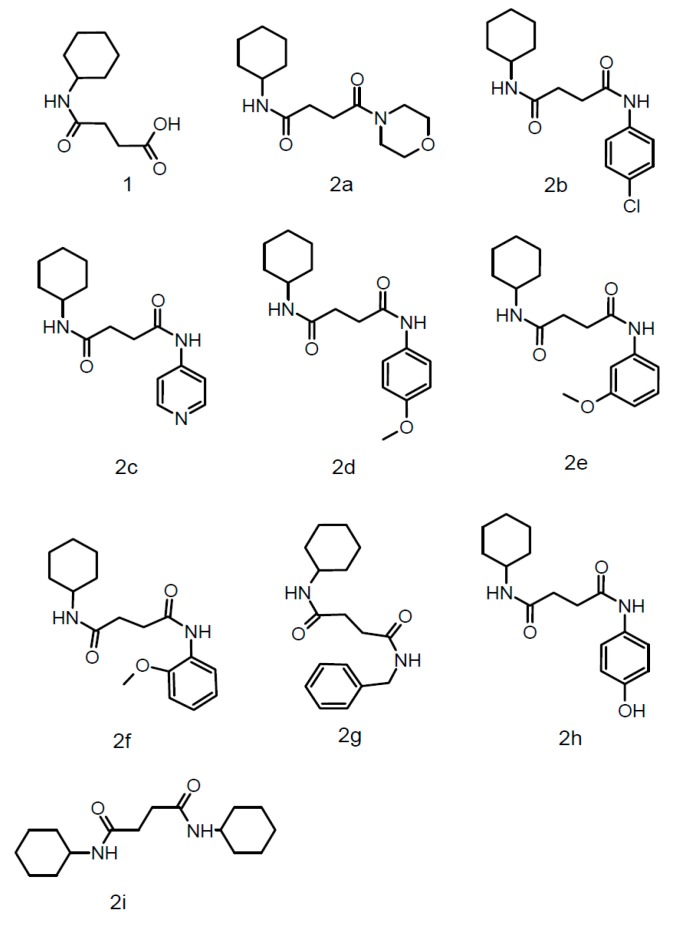
Structures of all newly synthesized succinamide derivatives.

Succinamide derivatives (2a–2i) were synthesized by dissolving equimolar quantities of succinic anhydride in dry dichloromethane and cyclohexylamine with continuous stirring at room temperature for 20 min while the progress of the reaction was monitored by TLC. After reaction completion, the solid separated was filtered and recrystallized from methanol to get the target compound 4-(cyclohexylamino)-4-oxobutanoic acid (parent compound) [21]. 1 mmol of carboxylic acid was added to 1 mmol of amine and 3 mmol of triethylamine (Et_3_N) in dichloromethane, then 1 mmol of SOCl_2_ was added at room temperature. The mixture was stirred continuously for 30–60 min at room temperature. The recovery of the reaction product was performed by evaporating the solvent under reduced pressure. The resulting residue was extracted in dichloromethane and washed first with 1 N hydrochloric acid (HCl) and then with 1 N sodium hydroxide (NaOH). The organic phase was dried by using anhydrous sodium sulphate (Na_2_SO_4_), recrystallized with methanol and evaporated to dryness to afford the corresponding carboxylic amide (Figure 1: general scheme; Figure 2: structures of newly synthesized succinamide derivatives).

### 2.3. DPPH Free Radical Scavenging Assay

The anti-oxidant potential of succinamide derivatives was measured by using 2,2-diphenyl-1-picrylhydrazyl (DPPH) free radical scavenging assay. Stock solution of test compounds and ascorbic acid (positive control)having concentration of 1 mg/mLwas prepared and further sample concentrations were prepared by serial dilution method (700, 300, 100, 10, 3, and 1 µg/mL). 1 mmol DPPH solution was prepared in methanol. Then, 1 mLof the test sample was taken from each dilution in separate test tubes and 3 mLof already prepared DPPH solution was added in each test tube to make up the final volume of 4 mL. All test tubes were covered with aluminum foil and kept at room temperature. In case of the presence of oxidation potential in test compounds, the inherently purple color of DPPH will change from purple to yellow due to free radical scavenging and absorbance will be recorded by using UV spectrophotometer at 517 nm. Percent inhibition or percent scavenging will be calculated by using the formula given as under [22].

Percentage scavenging activity = Absorbance of control − Absorbance of sample × 100 Absorbance of control

### 2.4. In-Silico Studies

The in-silico studies of newly synthesized succinamide derivatives were carried out to gain a perception of their potential binding affinities at the active binding sites of the target proteins via Autodock Vina version 4.2.6 (San Diego, CA, USA). The PDB files of X-ray crystal structures of selected receptors TNF-α (PDB ID: 2AZ5) and COX2 (PDB ID: 3ln1) were retrieved from the online protein database http://www.rcsb.org/pdb. Active binding pockets of targets were retrieved from https://bio.tools/dogsitescorer (DoG Site Scorer) [23]. Preparation of ligand-protein complexes was done for molecular docking. Biovia Discovery Studio Visualizer (DSV) was employed to clean the target proteins by removing water and cocrystallized ligands and saved as PDB format. Mol files of ligands were generated by drawing their structures in ChemSketch. All files of succinamide derivatives and ligands were transformed into PDB format via Open Babel [23]. Furthermore, PDBQT files of targets and ligands were prepared through AutoDock Tools version 1.5.6. Moreover, PyRx, digital software for molecular docking was used for docking purpose [24]. Biovia DSV was employed to interpret the best conformational poses and ligand-target molecular interactions. Validation of the docking procedure was carried out by comparing the best poses of the cocrystallized ligands with the best poses of re-docked configurations.

### 2.5. Animals and Experimental Groups

Balb-C mice of either gender (25–30g) were housed at controlled temperature (22–25 °C) and kept on a 12 h: 12 h light–dark cycle, with free access to food and water. Mice were group-housed throughout the testing period. All the animals were randomly divided into seven groups *(n* = 10 in each group): (i) saline-treated control group, containing 2.5% DMSO (10 mL/kg, i.p); (ii) scopolamine (SCO) treated disease group (1 mg/kg, i.p); (iii)–(vii) compound **2b**, **2d**, **2e**, **2g** and **2i** (250 ug/kg, i.p) + SCO. All compounds were initially solubilized in 2.5% dimethyl sulfoxide (DMSO) and volume make up was done with saline. All experiments were carried out in accordance with the approved protocols of the research and ethical committee (REC) of Riphah Institute of pharmaceutical science (RIPS), Riphah International University, Islamabad, Pakistan (REC/RIPS/2018/17, approved on 18-07-2018). After behavioral studies, animals were sacrificed employing standard protocol using CO_2_ euthanasia. For one cohort, brain tissues were extracted and stored at −80 °C followed by tissues homogenization to collect the supernatant for further analysis (*n* = 5). For another cohort, brain tissues were fixed in 4% formalin, later embedded in paraffin, 4 µm thin coronal sections were made by a rotary microtome (*n* = 5).

### 2.6. Behavioural Studies

#### 2.6.1. Y-Maze Test

Spatial working memory was measured by the Y-maze test. Y-maze is a three-arm horizontal maze (50 cm long and 10 cm wide) with walls 20 cm high and the arms are symmetrically inclined at 120° to each other. Mice were divided into seven groups (*n* = 10) and received a single dose per day for four consecutive days. Group (i) was used as control and received an i.p dose of mixture of saline and 2.5% DMSO (10 mL/kg), Group (ii) was used as disease group which received an i.p dose of SCO (1 mg/kg), in Groups (iii)–(vii) (disease + treatment), one hour before the test, mice were treated with 250 µg/kg dose of test compound i.p and after 30 min SCO (1 mg/kg) was administered. Briefly, animals were set free for spontaneous movement throughout the Y-maze by placing them at the middle point of the maze. Animal entries into different arms were recorded by using a digital camera. Each of the un-interrupted entry was considered to be spontaneous alteration behavior [25]. The percentage of alteration was determined by the following equation:

% Alteration = ((Number of alterations)/(Total arm entries−2)) × 100

The extent of neurodegeneration was estimated by elevation in percent spontaneous alteration behavior.

#### 2.6.2. Morris Water Maze Test (MWM)

Mice were distributed into seven groups (*n* = 10) and received a single dose per day for four consecutive days. Group (i) was used as control which received an i.p dose of mixture of saline and 2.5% DMSO (10 mL/kg), Group (ii) was used as disease group which received an i.p dose of SCO (1 mg/kg), in Groups (iii)–(vii) (disease + treatment), one hour before the test, mice were treated with 250 µg/kg dose of test compound i.p and 30 min after SCO (1 mg/kg) was administered. Mice were subjected to four trials per day for four consecutive days (a minimum of 15 min difference were maintained between each trial) with the platform in place. Once the mice located the platform, it was permitted to remain there for 10s. If the mouse was incapable to locate the platform within 60 s, it was positioned to the platform and stayed for 10s and then removed from the pool. On the fifth day of the MWM, each mouse was individually subjected to a probe trial session and mice were probed for time spent in the platform quadrant. Mice were allowed to swim for 60s and escape latency time was recorded by a video camera [26].

### 2.7. Hematoxylin Eosin (H&E) Staining

After de-paraffinizing tissue slides using absolute xylene (100%), it was followed by rehydrating with absolute ethanol, gradient ethanolic concentrations (95% to 70%), and subsequently with distilled water. Slides were then rinsed with PBS and were kept in hematoxylin for a total of 10 min. Then slides were placed for 5 min under running tap water in a glass jar. Slides were then probed under the microscope for nuclear staining, and if staining was not clear, hematoxylin timing was increased. Slides were then treated with 1% HCl and 1% ammonia water for a short interval and immediately rinsed with water again. Slides were then immersed in eosin solution for 5–10 min, followed by rinsing with water and finally air-dried [27]. Slides were then dehydrated using graded ethanol (70%, 95%, and 100%), fixed in xylene and cover-slipped. Images were captured using an Olympus light microscope (Olympus, Shinjuku, Tokyo, Japan) and analyzed using ImageJ software. Five images per group were captured and analyzed while focusing on neuronal shape, size, infiltrated cells, and vacuolation.

### 2.8. Immunohistochemical Analysis

Immunohistochemical analysis was performed as described previously with minor modifications [28]. After deparaffinization and rehydration procedures, slides were subjected to an enzymatic method for antigen retrieval step using proteinase K. Slides were then washed using 0.1 M PBS, and endogenous peroxidase activity was quenched with 3% hydrogen peroxide for 10 min. Again, slides were washed with 0.1 M PBS and incubated with 5% NGS (normal goat serum) containing 0.1% Triton X-100 for a minimum of 1 h in a humidified chamber. After blocking, slides were kept for overnight incubation at 4 °C with primary antibodies as anti-COX-2, and anti-TNF-α(Dilution 1: 100, Santa Cruz Biotechnology, Dallas, Texas, USA). The next morning, after washing twice with 0.1 M PBS, they were incubated for 90 min with biotinylated secondary antibodies (dilution factor 1:50) in a humidified chamber. Slides were again washed and incubated for 1 h with ABC reagents (Santa Cruz Biotechnology, Dallas, Texas, USA) in a humidified chamber. Slides were then stained in DAB solution, washed with distilled water, dehydrated in graded ethanol, fixed in xylene and cover-slipped using mounting medium. Immunohistochemical TIF images of the slides were taken using a light microscope (Olympus, Shinjuku, Tokyo, Japan) taking three images per slide. Hyper activated TNF-α and COX-2 were quantified using Image J software and expressed in terms of the relative integrated density of the samples comparative to the control.

### 2.9. Assessment of Antioxidant Enzymes

#### 2.9.1. GSH and GST Analysis

Previously documented assay protocols were employed for measuring glutathione (GSH) and glutathione-S-transferase (GST) levels. Pre-homogenized samples were treated with PBS followed by the addition of 2-nitrobenzoic acid solution. Absorbance values were measured at a wavelength of 412 nm. Similarly, GST levels were also measured by using previously used assay protocol with slight modification. Briefly, equimolar concentrations of 1-chloro-2,4-dinitrobenzene and glutathione-S-transferase were added together and after thorough mixing was diluted with 0.1 M PBS. A wavelength of 340 nm was set to take absorbance values of sample homogenate following serial dilution [28].

#### 2.9.2. LPO Assay

Lipid peroxidation (LPO) assay was carried out by employing previously used protocol with slight modification [29]. Lipid peroxidation was measured in terms of thiobarbituric acid reactive substances (TBARS) in tissue sample homogenates of mice.

#### 2.9.3. Catalase Assay

Catalase assay was done in accordance with the specifications as mentioned before with minor modifications [30]. A quantity of 10 μL of the sample was added to each well followed by the addition of 290 μL of 3% H_2_O_2_. Then, the96-well plate was stored at room temperature in darkness for 10 min and absorbance was measured at a wavelength of 440 nm.

### 2.10. Enzyme-Linked Immunosorbent Assay (ELISA)

Enzyme-linked immunosorbent assay for COX2, p-NF-κB, and TNF-α was carried out in accordance with the manufacturer’s instructions (Shanghai Yuchun Biotechnology, Shanghai, China). Briefly, brain tissues stored at −80°C were homogenized and the supernatant was collected following centrifugation (at 13,500× *g* for 1 h). p-NF-κBElisa kit (Cat # SU-B28069), and TNF-α Elisa kit (Cat. # SU-B3098) were purchased from (Shanghai Yuchun Biotechnology, Shanghai, China). Briefly, the protein samples were reacted with respective antibodies provided in the kit using a 96-well plate and absorbance values were measured via microplate reader, BioTekEL x808. Results were expressed as a concentration in picograms per milli Liter (pg/mL). All steps were performed in triplicate.

### 2.11. Statistical Analysis

Results were expressed as mean ± SEM and analysis was done by applying one-way analysis of variance (ANOVA) followed by post hoc Tuckey’s test through Graph Pad Prism 6 (San Diego, CA, USA). Moreover, the behavioral data was analyzed by two-way grouped analysis. Histological data were analyzed through ImageJ software. Symbol # denotes significance against the saline group and *denotes significance against the scopolamine group. Data were considered to show statistical significance at a value of *p* < 0.05.

## 3. Results

### 3.1. Spectral Analysis

#### 3.1.1. 4-(Cyclohexylamine)-4-oxobutanoic acid (**1**)

White solid: yield 89% m.p (observed):155–157 °C; ^1^H-NMR (DMSO-d6, 300 MHz, δ ppm): 1.08–1.71 (m, 11H, cyclohexyl-H), 2.27(t, *J* = 6.9 Hz, −CH_2_), 2.50(t, *J* = 6.5 Hz,−CH_2_), 7.12 (s, 2H, amide-NH), 11.99 (s, 1H, −COOH).

#### 3.1.2. *N*-cyclohexyl-4-(morpholin-4-yl)-4-oxobutanamide (**2a**)

Light brown solid; yield 25%; m.p 180–182°C; FTIR cm^−1^: 3292(N-H), 1660(C=O), 2851(C-H); ^1^H-NMR (DMSO-d6, 300 MHz, δ ppm): 1.22–1.72(m, 11H, cyclohexyl-H), 2.49 (t, 2H, *J* = 7.2 Hz, -CH_2_), 2.89(t, 2H, *J* = 7.4 Hz, -CH_2_), 3.33–3.52 (m, 8H, morpholin-H), 7.62 (s, 2H, amide-NH); elemental analysis: C_14_H_24_N_2_O_3_; calculated: C 62.60%, N 10.43%, H 8.94%, found: C 63.17%, N 9.94%, H 9.05%.

#### 3.1.3. *N*-(4-chlorophenyl)-*N*′-cyclohexylbutanediamide (**2b**)

Pale white solid; yield 76%; m.p 210–213 °C; FTIR cm^−1^: 3295(N-H), 1665(C=O), 2854(C-H); ^1^H-NMR (DMSO-d6, 300 MHz, δ ppm): 1.09–1.72(m, 11H, cyclo-H), 2.37 (t, 2H, *J* = 7.4 Hz, -CH_2_), 2.53(t, 2H, *J* = 7.3 Hz, -CH_2_), 7.34–7.62 (m, 4H, Aryl-H), 8.31 (s, 1H, amide-NH), 10.09 (s, 1H, amide-NH); elemental analysis: C_16_H_21_N_2_O_2_Cl; calculated: C 62.17%, N 9.06%, H 6.80%;found: C 62.50%, N 9.00%, H 7.13%.

#### 3.1.4. *N*-cyclohexyl-*N*′-(pyridin-4-yl)butanediamide) (**2c**)

Dark maroon solid; yield 25%; m.p 125–127 °C; FTIR cm^−1^:3296(N-H), 1685(C=O), 2927(C-H); ^1^H-NMR (DMSO-d6, 300 MHz, δ ppm):1.36–1.74 (m, 11H, cyclo-H), 2.60(t, 2H, *J* = 7.3 Hz, -CH_2_), 2.91(t, 2H, *J* = 7.2 Hz, -CH_2_), 7.35–8.51(m, 4H, pyridine-H), 9.64(s, 1H, amide-NH), 11.48 (s, 1H, amide-NH); elemental analysis: C_15_H_21_N_3_O_2_; calculated: C 65.37%, N 15.25%, H 7.62%; found: C 66.12%, N 15.59%, H 7.92%.

#### 3.1.5. *N*-cyclohexyl-*N*′-(4-methoxyphenyl)butanediamide) (**2d**)

Off-white solid; yield 55%; m.p 180–182°C; FTIR cm^−1^: 3271(N-H), 1661(C=O), 2924(C-H); ^1^H-NMR (DMSO-d6, 300 MHz, δ ppm): 1.19–1.14(m, 11H, cyclo-H), 2.59 (t, 2H, *J* = 7.2 Hz,-CH_2_), 2.70(t, 2H, *J* = 7.4 Hz, -CH_2_), 3.79(s, 3H, -OCH_3_), 5.77 (d, 1H, *J* = 6.6 Hz), 7.45–6.83 (m, 4H, Aryl-H), 8.31(s, 1H, amide-NH); elemental analysis: C_17_H_24_N_2_O_3_; calculated: C 67.02%, N 9.19%, H 7.88%; found: C 67.13%, N 8.98%, H 8.19%.

#### 3.1.6. *N*-cyclohexyl-*N*′-(3-methoxyphenyl butanediamide) (**2e**)

Dark brown solid; yield 56%; m.p 130–133 °C; FTIR cm^−1^: 3296(N-H), 1664(C=O), 2920(C-H); ^1^H-NMR (DMSO-d6, 300 MHz, δ ppm): 1.10–1.45 (m, 11H, cyclohexyl-H), 2.68 (t, 2H, *J* = 7.2 Hz, -CH_2_), 2.84 (t, 2H, *J* = 7.3 Hz, -CH_2_), 3.70 (s, 3H, -OCH_3_), 6.96–7.62 (m, 4H, Aryl-H), 7.11(s, 1H, amide-NH), 8.89(s, 1H, amide-NH); elemental analysis: C_17_H_24_N_2_O_3_; calculated: C 67.02%, N 9.19%, H 7.88%; found: C 66.91%, N 9.20%, H 7.51%.

#### 3.1.7. *N*-cyclohexyl-*N*′-(2-methoxyphenyl)butanediamide) (**2f**)

Light yellow solid; yield 38% 140–142 °C FTIR cm^−1^:3314(N-H), 1669(C=O), 2851(C-H); ^1^H-NMR (DMSO-d6, 300 MHz, δ ppm): 1.29–1.87(m, 11H, cyclohexyl-H), 2.38(t, 2H, *J* = 7.4 Hz, -CH_2_), 2.94(t, 2H, *J* = 7.5 Hz, -CH_2_),3.68(s, 3H,-OCH_3_),), 7.17-7.33 (m, 4H, Aryl-H),9.86 (s, 1H, amide-NH), 11.42 (s, 1H, amide-NH); elemental analysis: C_17_H_24_N_2_O_3_; calculated: C 67.02%, N 9.19%, H 7.88%; found: C 67.23%, N 9.31%, H 7.05%.

#### 3.1.8. *N*-benzyl-*N*′-cyclohexylbutanediamide (**2g**)

Yellow solid; yield 66% 180–182 °C; FTIR cm^−1^: 3285(N-H), 1662(C=O), 2921(C-H); ^1^H-NMR (DMSO-d6, 300 MHz, δ ppm 1.41–1.77(m, 11H, cyclohexyl-H), 2.60(t, 2H, *J* = 7.4 Hz, -CH_2_), 2.79(t, 2H, *J* = 7.4 Hz, -CH_2_), 4.50(s, 2H, -CH_2_), 7.23–7.86(m, 4H, Aryl-H), 7.19(s, 1H, amide-NH), 7.91(s, 1H, amide-NH); elemental analysis: C_17_H_24_N_2_O_2_; calculated: C 70.7%, N 9.70%, H 8.32%; found: C 71.06%, N 9.60%, H 7.95%.

#### 3.1.9. *N*-cyclohexyl-*N*′-(4-hydroxyphenyl)butanediamide **(2h**)

Off-white solid; yield 30% 155–156°C; FTIR cm^−1^: 3309(N-H), 1675(C=O), 2855(C-H); ^1^H-NMR (DMSO-d6, 300 MHz, δ ppm): 1.40–1.79(m, 11H, cyclohexyl-H), 2.69(t, 2H, *J* = 7.3 Hz, -CH_2_), 2.86(t, 2H, *J* = 7.4 Hz, -CH_2_), 6.36(s, 1H, OH), 6.61–7.13(m, 4H, Aryl-H),7.84(s, 1H, amide-NH), 8.47(s, 1H, amide-NH); elemental analysis: C_16_H_22_N_2_O_3_; calculated: C 66.12%, N 9.64%, H 7.57%; found: C 66.51%, N 9.35%, H 8.01%.

#### 3.1.10. *N*,*N*′-di cyclohexyl butanediamide (**2i**)

White solid; yield 68% 140–142°C; FTIR cm^−1^:3284(N-H), 1670 (C=O), 2853(C-H); ^1^H-NMR (DMSO-d6, 300 MHz, δ ppm): 1.56–1.68(m, 11H, cyclohexyl-H), 1.70–1.89(m, 11H, cyclohexyl-H), 2.62(t, 2H, *J* = 7.1 Hz, -CH_2_), 2.76(t, 2H, *J* = 7.3 Hz, -CH_2_), 7.14(s, 1H, amide-NH), 8.46 (s, 1H, amide-NH); elemental analysis: C_16_H_28_N_2_O_2_; calculated: C 68.47%, N 9.98%, H 9.98%, found: C 69.05%, N 8.95%, H 10.13%.

### 3.2. Effect of Succinamide Derivatives on DPPH Free Radical Scavenging Assay

**Figure 3:**
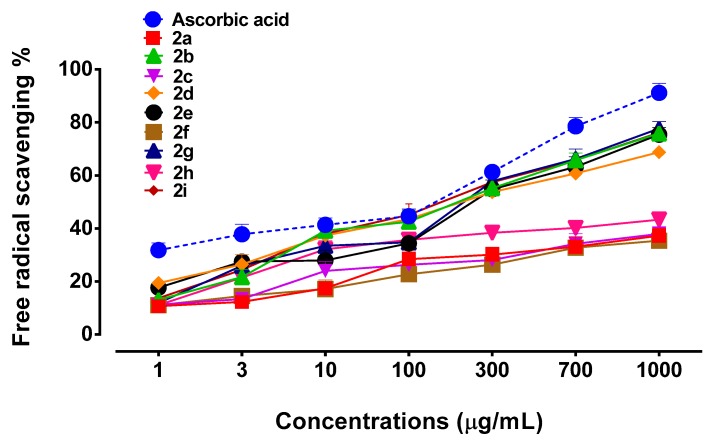
Illustrates percent free radical scavenging of novel succinamide derivatives (**2a–2i**) and ascorbic acid against 2,2-diphenyl-1-picrylhydrazyl (DPPH) free radical. Values are expressed as mean ± SEM.

As illustrated in Figure 3 compounds **2g**, **2i**, **2d**, **2b**, **and 2e** exhibited significant antioxidant potential when compared to commercially available reference standard ascorbic acid. Although compounds **2a**, **2c**, and **2h** also demonstrated antioxidant activity to some extent, it was not comparable with ascorbic acid or other succinamide derivatives. The order of antioxidant ability of succinamide derivatives was found to be ascorbic acid > compound **2e** > compound **2i** > compound **2b** > compound **2g** > compound **2d** > compound **2c** > compound **2f** > compound **2a** > compound **2h** (Figure 3). Five newly synthesized succinamide derivatives (**2b**, **2d**, **2e**, **2g**, and **2i**) relatively exhibited a higher antioxidant potential and were selected for further in vivo studies.

### 3.3. Evaluation of In-silico studies

Molecular docking of five newly synthesized succinamide derivatives (**2a**–**2i**) and cocrystallized ligands were carried out against active binding sites of TNF-α and COX2. The results obtained are illustrated in Table 1.

**Figure 4:**
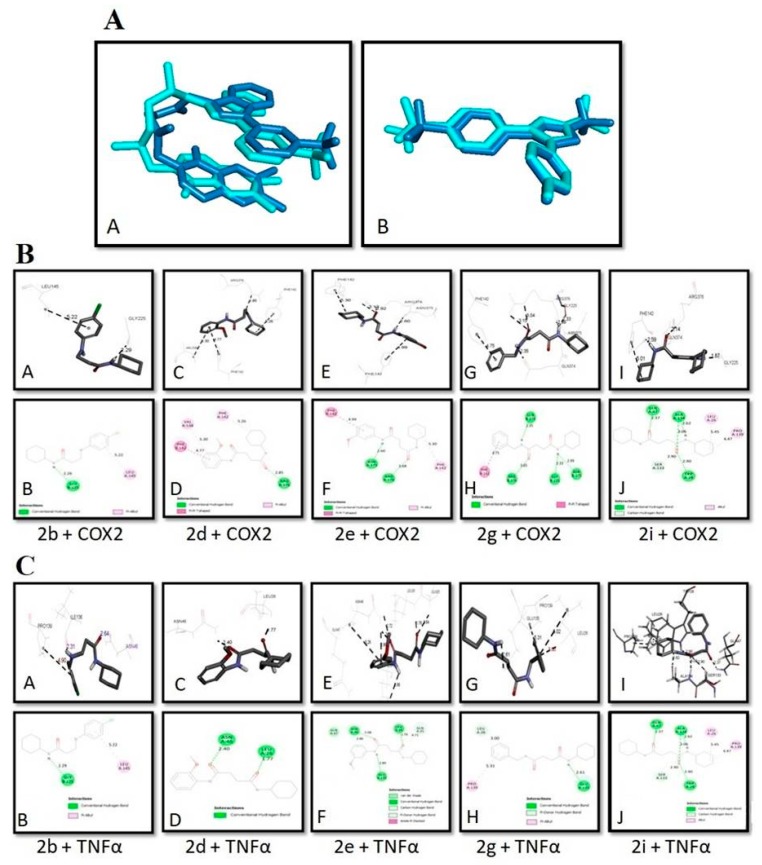
Best binding pose for 6,7-dimethyl-3-[(methyl{2-[methyl({1-[3(trifluoromethyl)phenyl]-1h-indol-3-yl} methyl)amino]ethyl}amino)methyl]-4h-chromen-4-one and celecoxib in the binding pockets of and tumor necrotic factor (TNF-α) and cyclooxygenase-2 (COX-2) respectively (dark-blue = co-crystallized ligand, cyan = re-docked ligand) (**A**,**B**).(**B**) Post dock analysis performed through Biovia Discovery StudioVisualizer illustrating both 2D and 3Dposes. Interactions between **2b** and COX2 (**A**,**B**), **2d** and COX2 (**C**,**D**), **2e** and COX2 (**E**,**F**), **2g** and COX2 (**G**,**H**), and **2i** and COX2 (**I**,**J**). (**C**)Post docking analysis visualized by Discovery Studio Visualizer in both 2D and 3D styles. Interactions between **2b** and TNF-α (**A**,**B**), **2d** and TNF-α (**C**,**D**), **2e** and TNF-α (**E**,**F**), **2g** and TNF-α (**G**,**H**), and **2i** and TNFα (**I**,**J**) respectively.

Cocrystallized ligands were re-docked against the same target protein in order to validate the procedure being employed for molecular docking. A comparison of the best binding poses generated before and after re-docking of cocrystallized molecules against TNF-α and COX2 are illustrated in Figure 4A (A,B). The 2D and 3D images of the interaction of succinamide derivative **2b** with COX2 are illustrated in Figure 4B (A,B). Succinamide derivative **2b** possesses an amide bond which established a hydrogen bond with Gly B:225 residue with a bond distance of 2.29 Å, while chloro benzyl group made hydrophobic contacts with Leu A:145. Figure 4B (C,D) illustrates the binding pose of compound **2d** with COX2. The carbonyl group was stabilized by hydrogen bond interactions with ARG A:376 with a bond distance of 2.85 Å and π–alkyl and π–π T-shaped contacts were observed with o–anisidine ring involving Val A:538 and Phe A:142 residues respectively. Another π–alkyl interaction was observed between the cyclohexyl group and Phe A:142 residue. The binding modes and molecular interactions with the lowest energy of succinamide derivative **2e** with COX2 are represented in Figure 4B (E,F). The N-H and carbonyl groups in amide were stabilized by forming a hydrogen bond with Asn A:375 and Arg A:376 with a bond distance of 2.60 Å and 3.68 Å respectively. The cyclohexylamine scaffold exhibited a π–alkyl bond with PheA:142 and m-anisidine moiety interacted with Phe A:142 by π–π T-shaped interaction. Ligand–receptor interaction of succinamide derivative **2g** with COX2 is demonstrated in Figure 4B (G,H). In this case, 3 hydrogen bonds were depicted with NH of the amide bond involving binding residues of Gln B:374, Gly B:225 and ASN B:375 with a bond distance of 2.35, 2.33, and 2.99 Å respectively. Another hydrogen bond of Arg B:376 was established to stabilize the carbonyl group of amide (3.65 Å). Binding mode and interaction of succinamide derivative **2i** with COX2 are shown in Figure 4B (I,J). The binding interaction of succinamide derivative **2i** against COX2 demonstrated four H bonds, three between the Gln A:47, Ala A:134 and Trp A:28, and carbonyl group (2.37, 3.06, and 2.90 Å) and the fourth between the NH group and Ala A:134 (2.62 Å). The 2D and 3D images of the interaction of succinamide derivative **2b** with TNF-α are shown in Figure 4C (A,B). Succinamide derivative **2b** possesses an amide bond which formed a hydrogen bond with Gly B:225 (2.29 Å), while chloro benzylamine group made hydrophobic contacts with Leu A:145. Figure 4C (C,D) illustrates the binding pose of compound **2d** with TNF-α. Two carbonyl groups of the amide were stabilized by very strong hydrogen bond interactions with Leu A:26 and Asn A:46 (1.77 and 2.4 Å). The binding interaction of succinamide derivative **2e** against TNF-α is illustrated in Figure 4C (E,F). N–H and Carbonyl groups in amide were stabilized by establishing hydrogen bond with Asn A:46, Leu A:26, and Glu A:135 (5.08, 1.76, and 2.89 Å) respectively. The m-anisidine scaffold exhibited a hydrogen bond with Asn A:46 (2.86 Å). Ligand-target binding of succinamide derivative **2g** against TNF-α is represented in Figure 4C (G,H). In this case, 1 hydrogen bond was established with NH of the amide bond involving binding residues of Glu A:135 with a bond distance of 2.61 Å. The molecular interaction of succinamide derivative **2i** with TNFα is represented in Figure 4C (I,J). The binding mode of **2i** with TNF-α demonstrated four H bonds, three between the carbonyl group and Gln A:47, Ala A:134, and Trp A:28 (2.37, 3.06, and 2.90 Å)and the fourth between the NH group and Ala A:134 (2.62 Å).The cyclohexylamine moiety was further stabilized by hydrophobic interactions.

### 3.4. Effect of Succinamide Derivatives on Alteration Behaviour

**Figure 5:**
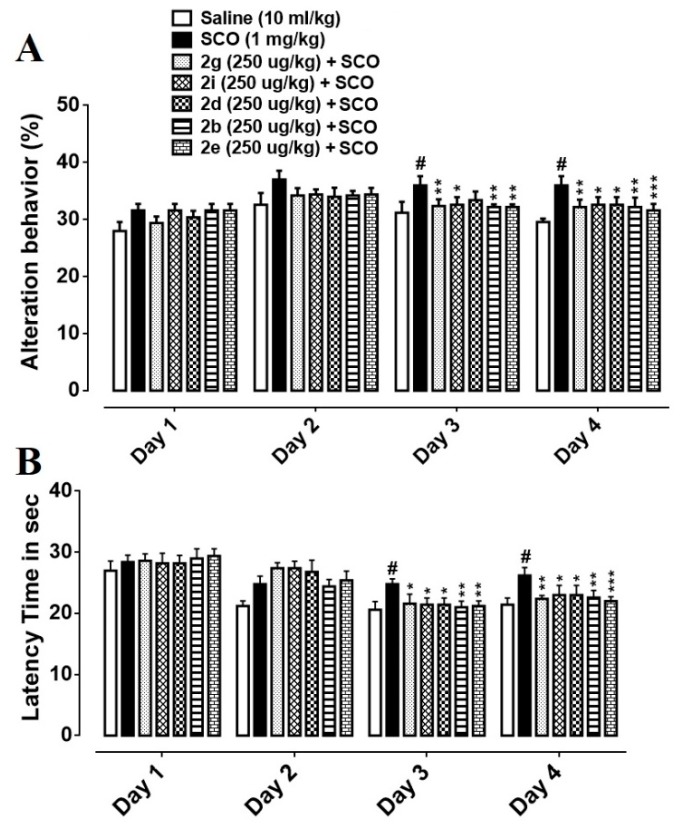
(**A**) % alteration behavior. Data were analyzed by mean±SEM with (*n* = 10). # denotes a significant difference against the saline group; *, **, *** show significant difference against the scopolamine (SCO) group. *p* < 0.05 is considered to be significant. (**B**) Average escape latency time. Mean±SEM for the mice (*n* = 10). # denotes a significant difference against the saline group; *, **, *** show significant difference against the scopolamine (SCO) group. *p* < 0.05 is considered to be significant.

The Y-maze task was performed to analyze the spatial working memory using spontaneous alteration behavior (%). The entire series of selected synthesized compounds showed increased spontaneous alteration behavior (%) in the Y-maze test. Alteration behavior (%) of SCO (1 mg/Kg) treated group showed significant difference against saline control in all trials carried out on 4 consecutive days (^###^ *p* < 0.001 vs. saline group). Alteration behavior (%) of SCO+**2g** (250 µg/Kg) group were not significantly reduced on day 1 and 2 but exhibited a significant decrease of * *p* < 0.05 vs. SCO group on day 3 and ** *p* < 0.01 vs. SCO group on day 4, respectively (Figure 5A). Alteration behavior (%) of SCO+**2i** (250 µg/Kg) group were insignificant on day 1 and 2 in comparison to scopolamine treated group but revealed a significant reduction of * *p* < 0.05 vs. SCO group on day 3 and * *p* < 0.05 vs. SCO group on day 4, respectively (Figure 5A). Alteration behavior (%) of SCO+**2b** (250 µg/Kg) group did not show marked difference from disease group on day 1 and 2, but demonstrated a significant difference of ** *p* < 0.01 vs. SCO group on day 3 and ** *p* < 0.01 vs. SCO group on day 4, respectively (Figure 5A). Alteration behavior (%) of the SCO+**2e** (250 µg/Kg) group were not significantly decreased on day 1 and 2 but showed marked decrease of ** *p* < 0.01 vs. SCO group on day 3 and *** *p* < 0.001 vs. SCO group on day 4, respectively (Figure 5A). Alteration behavior (%) of the SCO+**2d** (250 µg/Kg) group were again insignificant on day 1 and 2 but exhibited decreased alteration behavior (%) of * *p* < 0.05 vs. SCO group on day 3 and * *p* < 0.05 vs. SCO group on day 4, respectively (Figure 5A). Hence, significantly decreased % alteration behavior in succinamide derivatives treated mice indicates a reversal of scopolamine-induced learning and behavioral deficit.

### 3.5. Effect of Succinamide Derivatives on Escape Latency Time

In the MWM test, escape latency time for the newly synthesized compounds was measured in four trials per day for four consecutive days. Latency time of SCO (1 mg/Kg) treated group was insignificant on day 1 as compared to the saline group but showed noticeably increased latency time with a significance of ^##^ *p* < 0.01 vs. saline group, ^###^ *p* < 0.001 vs. saline group and ^###^ *p* < 0.001 vs. saline group on day 2, 3, and 4 respectively (Figure 5B). SCO+**2g** (250 µg/Kg) group exhibited no significant decrease of latency time on day 1 and 2, however, a markedly reduced escape latency time was observed on day 3 (* *p* < 0.05 vs. SCO group) and 4 (** *p* < 0.01 vs. SCO group), respectively (Figure 5B). The latency time of the SCO+**2i** (250 µg/Kg) group was found to be significantly decreased on day 3 (* *p* < 0.05 vs. SCO group) and day 4 (* *p* < 0.05 vs. SCO group) respectively (Figure 5B). The SCO+**2d** (250 µg/Kg) group demonstrated no significant reduction of latency time on day 1 and 2, but decreased escape latency was found on day 3 (* *p* < 0.05 vs. SCO group) and day 4 (* *p* < 0.05 vs. SCO group) respectively (Figure 5B). The latency time of the SCO+**2b** (250 µg/Kg) group was decreased on day 3(** *p* < 0.01 vs. SCO group) and day 4 (** *p* < 0.01 vs. SCO group) respectively. A significant reduction of latency time was seen in the SCO+**2e** (250 µg/Kg) group on day 3 (** *p* < 0.01 vs. SCO group) and day 4 (*** *p* < 0.001 vs. SCO group) respectively (Figure 5B). Therefore, we may perceive that this significant decrease in escape latency time in succinamide derivatives treated groups indicate a reversal of scopolamine-induced cognitive deficit.

### 3.6. Effect of Succinamide Derivatives on Scopolamine-Induced Neurodegeneration

**Figure 6:**
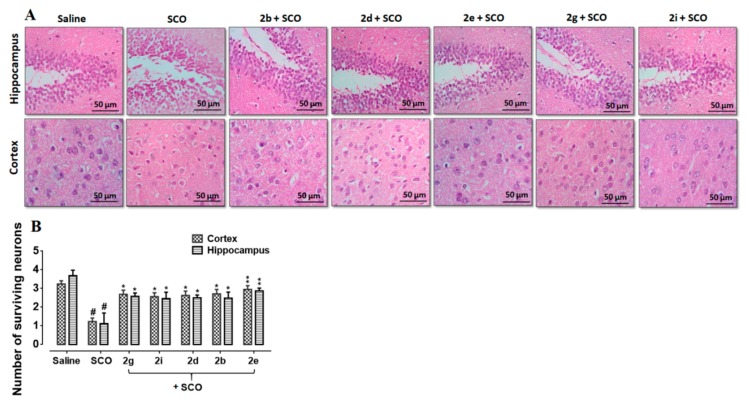
H&E staining showing the extent of surviving neurons in the cortex and hippocampus (dentate gyrus, dg). Bar 50 μm, magnification 40×, *n* = 5 group. Surviving neurons were marked by cytoplasmic swelling, vacuoles, scalloped neurons with intense cytoplasmic eosinophilia, and nuclear basophilia. These changes resulted from neuronal necrosis. Some cells had a shrunken appearance, along with pyknotic nuclei (**A**). # shows significant difference relative to saline; * shows significant difference relative to scopolamine. Data presented as means ± SEM. ** *p* < 0.01, * *p* < 0.05 (**B**).

Neuroprotective potential of succinamide derivatives was further elucidated by hematoxylin and eosin staining. The scopolamine treated group demonstrated vigorous histological changes inthe cortex and hippocampus of the brain of treated mice;as compared to the saline control group (Figure 5, *p* < 0.01). As illustrated, scopolamine administration elicited abnormal histological features including altered scalloped morphology of neurons associated with pyknosis, cytoplasmic eosinophilia, and nuclear basophilia. Succinamide derivatives administration demonstrated marked mitigation of scopolamine-induced neurodegeneration. Hence, a greater degree of cellular intactness and integrity was observed in succinamide derivatives treated groups (*p* < 0.05, Figure 6).

### 3.7. Succinamide Derivatives Mediated Downregulation of Neuroinflammation

**Figure 7:**
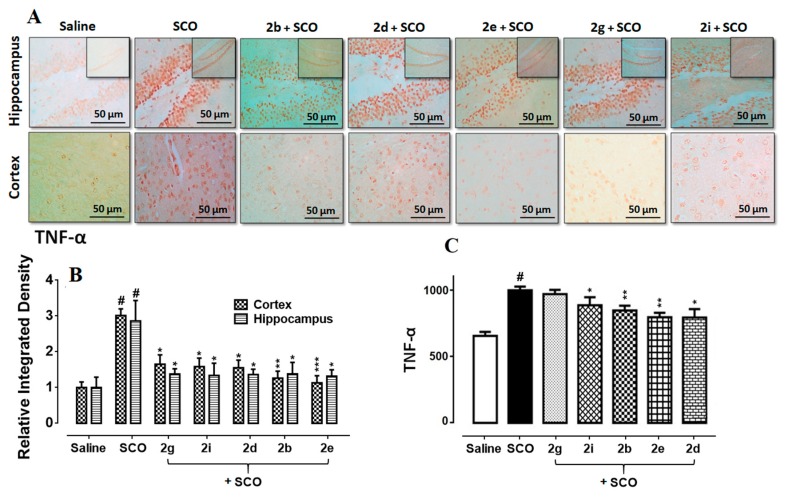
(**A**,**B**) Immunohistochemistry results for TNF-α in the cortex and hippocampus (dentate gyrus, dg). Bar 50 µm, magnification 40× (*n* = 5). Histograms show a comparatively higher expression of TNF-α in the scopolamine group. ***, ** and * show *p* < 0.001, *p* < 0.01 and *p* < 0.05, respectively, and illustrate significant variation relative to scopolamine, while ^###^ *p* < 0.001 shows significant variation relative to saline control group. Results are expressed as mean±SEM. (**C**) The protein expression of TNF-α quantified through ELISA. The data is presented as mean ± SEM. ^#^ *p* < 0.05 relative to saline control group, while * *p* < 0.05 and ** *p* < 0.01 relative to scopolamine (*n* = 5).

Marked elevation in the expression of TNF-α reactive cells has been observed in the scopolamine treated hippocampus and cortex comparative to saline control (Figure 7B, *p* < 0.001), followed by further validation through ELISA (Figure 7A, *p* < 0.05). Significant attenuation of these detrimental effects was carried out by the administration of succinamide derivatives (Figure 7, *p* < 0.05). 

### 3.8. Succinamide Derivatives Attenuated the Scopolamine-Induced Inflammatory Mediators

**Figure 8:**
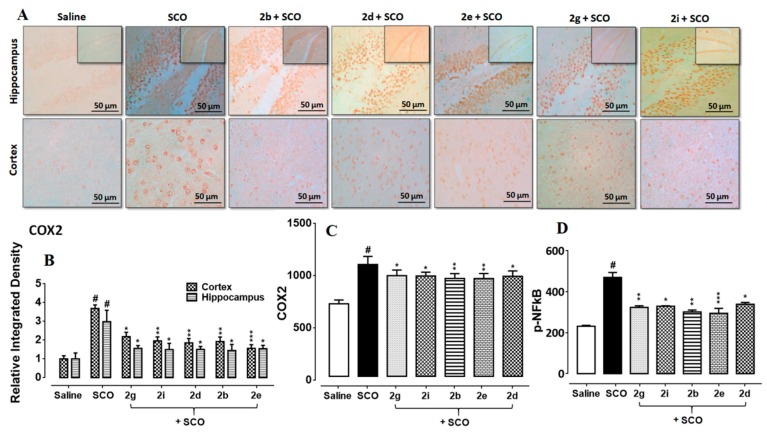
(**A**,**B**) Immunohistochemistry results for COX2 in the cortex and hippocampus (dentate gyrus, dg). Bar 50 µm, magnification 40× (*n* = 5). Histograms show a comparatively higher expression of COX2 in the scopolamine group. ***, ** and * show *p* < 0.001, *p* < 0.01, and *p* < 0.05, respectively, and illustrate significant variation relative to scopolamine, while ^###^ *p* < 0.001 shows significant variation relative to saline control group. Results are expressed as mean±SEM. (**C**) The protein expression of COX2 quantified through ELISA. The data is presented as mean±SEM. * *p* < 0.05 relative to scopolamine, while ^#^ *p* < 0.05 and ^##^ *p* < 0.01 relative to saline control group (*n* = 5). (**D**)The protein expression of p-NFkB quantified through ELISA. The data is presented as mean±SEM. * *p* < 0.05 relative to scopolamine, while ^#^ *p* < 0.05 and ^##^ *p* < 0.01 relative to saline control group (*n* = 5).

The interaction of inflammatory mediators with its designated targets results in sequential induction of downstream signaling pathways including JNK (c-Jun N-terminal kinase), SEK1 (extracellular signal-regulated kinase 1/stress-activated protein) and ASK1 (apoptosis signal-regulating kinase 1). The overall outcome is the proteosome detachment of IkB and NF-kB translocation into the nucleus to activate the inflammation triggering transcription machinery such as COX2 [5]. ELISA results confirmed the overexpression of COX2 and p-NFkB in the scopolamine treated brain (Figure 8A,B, *p* < 0.01). Succinamide derivatives demonstrated a noticeable decrease in COX2 overexpression (*p* < 0.05). Further validation was performed through immunohistochemical analysis and a similar pattern of COX2 expression was found in the scopolamine treated brain whereas succinamide derivatives significantly downregulated the COX2 levels (Figure 8C, *p* < 0.05).

### 3.9. Effect of Succinamide Derivatives on Antioxidant Enzymes

Table 2 illustrates the data obtained for scopolamine-induced antioxidant enzymes variation and its attenuation carried out by succinamide derivatives administration. Scopolamine treatment caused the accumulation of ROS and reduced the levels of antioxidant enzymes including catalase, GSH, and GST in the brain (*p* < 0.001). Administration of succinamide derivatives produced significant mitigation of downregulated antioxidant enzymes as illustrated in Table 2.

### 3.10. Effect of Succinamide Derivatives on Scopolamine-Induced Lipid Peroxidation (LPO)

Multiple studies reported malondialdehyde (MDA) as a critical marker for the estimation of oxidative burden. Thiobarbituric acid reactive substances (TBARS) is frequently employed assay for assessment of malondialdehyde (MDA), an important final product of LPO. TBARS assay was conducted accordingly and data obtained are shown in Table 2. Succinamide derivatives (**2g**, **2i**, **2d**, **2b**, **and 2e**) significantly down regulated LPO level.

## 4. Discussion

Previous studies revealed that several substituted succinamic acids showed anti-inflammatory activity and antitumor properties [17]. In the present study, a series of novel succinamide derivatives were designed and synthesized. Molecular docking results against COX2 and TNF-α were obtained to rank and reveal their binding and atom interacting details. In vitro, free radical scavenging assay showed that all these compounds exhibited antioxidant ability in a dose-dependent way. Further in vivo results showed that new derivatives markedly reduced scopolamine-induced neuroinflammation, oxidative stress, and neuronal degeneration. Moreover, they have prominently ameliorated the cognitive deficits induced by scopolamine.

The last two decades witnessed the identification of multiple oxidative species and inflammatory mediators involved in the pathophysiology of neurodegenerative disorders [31], where cognition related hippocampus and cortex are adversely affected [32,33]. Accordingly, the administration of anti-inflammatory substances including COX2 inhibitors attenuates the inflammatory cascade and improves behavioral deficits [34]. Similarly, previous studies demonstrated the anti-amnesic activity of meloxicam and selegiline through the augmentation of endogenous antioxidant enzymes [35]. The succinamide moiety constitutes a vital part of several drugs and drug candidates [36]. A number of succinamide derivatives have been demonstrated as potential antioxidants, anti-inflammatory and neuroprotective agents [19,37,38]. Preliminary screening for the antioxidant potential of succinamide derivatives was carried out via in-vitro DPPH free radical scavenging assay. Five newly synthesized succinamide derivatives (**2b**, **2d**, **2e**, **2g**, and **2i**) relatively exhibited a higher antioxidant potential in preliminary screening and were selected for further in vivo studies. The antioxidant capacities of these derivatives could be attributed to their possible proton donor ability to neutralize the free radicals.

Neuroprotection by succinamide derivatives as demonstrated by the reduced neuronal loss was possibly mediated through their antioxidant and anti-inflammatory effects. Succinamide moiety relies on a basic scaffold “pyrrolidine-2,5-dione”, comprising of 5 membered rings containing a nitrogen atom with two carbonyl groups. Interestingly, frequently used natural/synthetic anti-Alzheimer’s drugs also possess an aromatic ring with a nitrogen atom and carbonyl group in their structure [19]. Whether these shared functional groups are responsible for the observed in vitro and in vivo effects warranted further structural and functional analysis.

For learning and memory deficit, Y maze test was used in which the percent alteration behavior indicates short term memory while the total number of arm entries represents general locomotor activity [39]. Results of % alteration behavior showed significant improvement of short term memory deficit in succinamide treated groups (Figure 5A). However, general locomotor activity (number of arm entries) did not show any improvement as no significant difference was observed in succinamide treated groups as compared to the scopolamine group (data not shown). These results were further corroborated by the Morris water maze test assessing hippocampus-dependent spatial learning aptitude [40,41], where succinamide derivatives caused considerable shortening of scopolamine-prolonged escape latency time (Figure 5B), which indicates an improvement in scopolamine-induced spatial memory impairment [40,42].

ROS induced oxidative stress is closely linked with neuroinflammation, which further exacerbates neurodegenerative disorders. Various inflammatory mediators and proinflammatory cytokines are involved in neuroinflammation such as TNF-α, COX2, interleukin 6, interleukin 10, inducible nitric oxide synthase, and nuclear factor kappa B [43,44]. Besides their free radical scavenging effects, neuroprotection by succinamide derivatives could also result from their augmentation on endogenous antioxidant proteins (GST, GSH, and catalase), as well as from amelioration of LPO and proinflammatory mediators. Taken together, our data supported the hypothesis that these drugs can attenuate the scopolamine-induced neuronal toxicity by modulating cytokines expression, inflammatory cascade, and antioxidant enzymes.

## 5. Conclusions

In conclusion, scopolamine-induced neurodegeneration activates various inflammatory mediators such as NFkB/COX2/TNF-α along with ROS mediated oxidative stress. Our newly synthesized succinamide derivatives ameliorated scopolamine-induced oxidative stress and inflammatory cascade, perhaps by regulating the ROS/TNF-α/COX2/NFkB pathway, ultimately providing neuroprotection against neuronal inflammation.

## Figures and Tables

**Table 1:** Binding energy values of docking. TNF, tumor necrosis factor; COX, cyclooxygenase.

	Binding Energies (kcal/mol)	
Compounds	COX2	TNFα
**2b**	−6.8	−5.5
**2d**	−7.6	−6.1
**2e**	−7.8	−6.0
**2g**	−7.9	−5.7
**2i**	−6.4	−5.9

**Table 2:** Effect of succinamide derivatives on antioxidant enzymes and lipid peroxidation.

Groups	GST (µmol/mg of Protein)	GSH (µmol CDNB Conjugate/min/mg of Protein)	Catalase (µmol H_2_O_2_/min/mg of Protein)	LPO (nmol/TBARS/mg of Protein)
Saline	88.25 ± 0.15	67.90 ± 0.56	165.6 ± 1.650	85.15 ± 0.91
Scopolamine	27.8 ± 2.89 ^###^	26.78 ± 2.78 ^###^	88.80 ± 3.890 ^###^	123.70 ± 3.71^###^
**2g**	45.1 ± 3.8 *	33.90 ± 1.23 *	125.1±3.230 **	87.45±8.90 **
**2i**	43.78 ± 3.7 *	32.78 ± 0.90 *	120.5 ± 8.890 *	96.89 ± 4.56 *
**2d**	44.10 ± 3.78 *	33.33 ± 0.67 *	127.4 ± 4.900 **	92.78 ± 2.78 *
**2b**	50.13 ± 3.89 **	35.33 ± 0.67 **	130.9 ± 6.890 **	85.12 ± 4.89 **
**2e**	55.23 ± 2.89 ***	37.23 ± 0.78 ***	135.9 ± 8.890 ***	78.89 ± 6.89 ***

Symbols *** or ### show significant difference at *p* < 0.001, while * and ** show significant difference at *p* < 0.05 and *p* < 0.01, respectively. The symbol * shows a significant difference relative to scopolamine and # shows significant difference relative to saline. Data are shown as mean± SEM (*n*=5). Abbreviations: GST, glutathione S-transferase; GSH, glutathione; TBARS, thiobarbituric acid reactive substances; LPO, lipid peroxidation; CAT, catalase.
